# From promise to practice: evaluating the clinical impact of FcRn inhibition in IgG-mediated autoimmune rheumatic diseases

**DOI:** 10.3389/fimmu.2025.1656937

**Published:** 2025-10-02

**Authors:** Chu Wei Yang, Ting Xia, Qing Tan, Li Gang Jie, Ai Ju Lou, Xiao Xiao Li, Maierhaba Maitiyaer, Wen Hui Huang, Yu Zheng, Shui Lian Yu

**Affiliations:** ^1^ Department of Rheumatology, The Second Affiliated Hospital, Guangzhou Medicial University, Guangzhou, Guangdong, China; ^2^ Department of Breast Surgery, The Second Affiliated Hospital, Guangzhou Medical University, Guangzhou, Guangdong, China; ^3^ Department of Rheumatology and Clinical Immunology, Zhujiang Hospital, Southern Medical University, Guangzhou, Guangdong, China; ^4^ Department of Rheumatology, Liwan Central Hospital of Guangzhou, Guangzhou, Guangdong, China; ^5^ Department of Urology, The Second Affiliated Hospital, Guangzhou Medical University, Guangzhou, Guangdong, China

**Keywords:** neonatal Fc receptor, IgG homeostasis, autoimmune diseases, FcRn inhibitors, efgartigimod

## Abstract

The neonatal Fragment Crystallizable receptor (FcRn) plays a central role in maintaining immunoglobulin (Ig) G homeostasis by protecting IgG from lysosomal degradation and regulating its transcytosis. In recent years, pharmacological inhibition of FcRn has emerged as a promising therapeutic strategy for IgG-mediated aumhctoimmune diseases, including systemic lupus erythematosus, rheumatoid arthritis, anti-Neutrophil Cytoplasmic Antibodies (ANCA) -associated vasculitis, and others. By accelerating the catabolism of circulating IgG, FcRn inhibitors effectively reduced pathogenic autoantibody levels without broadly suppressing other immune components. Several FcRn-targeting agents, such as efgartigimod, rozanolixizumab, and nipocalimab, have demonstrated favorable safety and efficacy profiles in clinical trials and are now approved or under investigation for multiple indications. This review also explored personalized therapeutic approaches, combination strategies, and the future landscape of FcRn-targeted drug development. While FcRn inhibition offered a paradigm shift in managing antibody-driven diseases, long-term safety and patient stratification remain key challenges for future research.

## 1 Introduction

Autoimmune diseases encompass a wide spectrum of disorders characterized by loss of immune tolerance and the production of pathogenic autoantibodies (1). Among these, IgG autoantibodies play a central role in mediating tissue damage in rheumatic conditions such as systemic lupus erythematosus (SLE), rheumatoid arthritis (RA), and ANCA-associated vasculitis (AAV). These antibodies form immune complexes, activate the complement cascade, and engage Fc gamma receptors, triggering inflammatory responses and progressive organ injury. Reducing pathogenic IgG has become a critical therapeutic goal in the management of such diseases.

Fc gamma receptors link IgG immune complexes to cellular effector programmes: activating FcγRI/IIA/IIIA trigger phagocytosis, antibody-dependent cell-mediated cytotoxicity (ADCC), and pro−inflammatory cytokine release by macrophages, neutrophils, and NK cells, whereas the inhibitory FcγRIIB dampens these signals. In autoantibody−driven diseases, overwhelming immune−complex load tips this balance toward activation, sustaining chronic inflammation and tissue injury.

The neonatal Fc receptor (FcRn), a non-classical Major Histocompatibility Complex (MHC) class I molecule, is essential for maintaining IgG and albumin homeostasis (2). It rescues IgG from lysosomal degradation through pH-dependent recycling and transcytosis. While this mechanism preserves antibody levels under physiological conditions, it also sustains circulating pathogenic IgG in autoimmune diseases. FcRn inhibitors block this recycling pathway, promoting IgG degradation and reducing serum IgG concentrations.

In recent years, multiple FcRn-targeting agents have entered clinical trials. Compounds such as efgartigimod, rozanolixizumab, and nipocalimab have demonstrated encouraging efficacy and safety in patients with IgG-mediated diseases, some gaining regulatory approval. With their selective mechanism and potential for rapid IgG clearance, FcRn inhibitors represent a novel and promising class of immunomodulatory therapy.

## 2 Neonatal Fc Receptor

### 2.1 Structure of FcRn

FcRn is a heterodimeric receptor composed of two subunits and shares structural homology with classical major histocompatibility complex class I (MHC I) molecules. It consists of a transmembrane heavy chain and a non-covalently associated light chain, β2-microglobulin (3). The heavy chain comprises three extracellular domains (α1, α2, and α3) and is encoded by the FCGRT gene. FcRn is only stably expressed and functionally active upon proper association with β2-microglobulin. Notably, FcRn has the unique ability to bind two distinct ligands—IgG and albumin—via independent and non-competing binding sites. This bifunctional binding enables FcRn to play multiple roles in immunoglobulin and protein homeostasis without competitive interference between the two ligands (4).

### 2.2 Distribution of FcRn

FcRn is widely distributed across various tissues in the human body (5). In the placenta, it is abundantly expressed in syncytiotrophoblasts, where it mediates the maternal-to-fetal transcytosis of IgG, ensuring passive immunity in the fetus. Additionally, FcRn is expressed in the parenchymal cells of several barrier tissues, including various epithelial cells and vascular endothelial cells. In a differentiated airway epithelium model (WD-HAE), FcRn has been shown to mediate the basal-to-apical transcytosis of IgG antibodies applied to the basolateral side, simulating systemic administration (6). In the intestinal epithelium, FcRn mediates bidirectional IgG transport between the lumen and the basolateral compartment through a pH-dependent mechanism, contributing to mucosal immune surveillance and maintenance of IgG homeostasis in the gut. In the renal glomerular filtration barrier, podocytes express FcRn, which is thought to prevent excessive retention of IgG and albumin, thereby protecting glomerular function. FcRn is also expressed in cells of the immune system, including mononuclear phagocytes, dendritic cells, and neutrophils. Its presence in these immune cells suggests a role in antigen presentation and the clearance of immune complexes. Collectively, the broad tissue distribution of FcRn underscores its physiological involvement in transcytosis, immunomodulation, and inter-organ homeostatic regulation, as elaborated in subsequent sections.

### 2.3 Regulatory mechanisms of FcRn expression

FcRn expression is modulated by various signaling molecules, with inflammatory cytokines playing a prominent role. Studies have shown that pro-inflammatory mediators such as TNF-α and Interleukin (IL)-1β upregulate FCGRT transcription and FcRn protein levels via activation of the Nuclear Factor kappa-light-chain-enhancer of activated B cells (NF-κB) signaling pathway (7). Conversely, Interferon (IFN)-γ suppresses FcRn expression by activating the Janus Kinase (JAK)/Signal Transducer and Activator of Transcription (STAT) 1 pathway. Specifically, IFN-γ-induced STAT1 can bind to interferon-stimulated response elements within the FCGRT promoter, resulting in transcriptional repression and decreased FcRn mRNA and protein levels (8). These cytokine-dependent regulatory pathways indicate that FcRn expression is dynamically modulated during immune responses, thus influencing systemic IgG and albumin homeostasis. In addition to inflammatory cues, FcRn expression exhibits developmental and tissue-specific regulation. For instance, FcRn levels in the human placenta increase progressively throughout gestation and peak in the late third trimester, which ensures maximal transfer of maternal IgG to the fetus near term.

### 2.4 Functions of FcRn

#### 2.4.1 Protection and recycling of IgG antibodies

IgG antibodies exhibit an extended serum half-life primarily due to FcRn-mediated recycling and protective mechanisms (5). Circulating IgG is continuously internalized by endothelial and other cells via pinocytosis into early endosomes. In the acidic milieu of these endosomes (pH ~6.0), FcRn binds specifically to the Fc region of IgG, diverting it from the lysosomal degradation pathway. Instead, FcRn–IgG complexes are sorted into recycling endosomes and transported back to the cell surface, where, upon exposure to the neutral extracellular environment (pH ~7.4), IgG dissociates from FcRn and is released back into the circulation. Through this intracellular trafficking loop, FcRn repeatedly salvages and recycles IgG, substantially prolonging its systemic persistence. As such, FcRn is considered a key “protective receptor” that maintains IgG homeostasis and prevents its premature catabolism. Structural studies have shown that under acidic conditions, the Fc region of a single IgG molecule can simultaneously bind two FcRn heterodimers, forming a stable complex that shields IgG from degradation.

#### 2.4.2 Protection of albumin

Albumin, the most abundant plasma protein, also enjoys a long half-life, largely attributed to FcRn-mediated recycling. Like IgG, albumin is continuously taken up by cells such as vascular endothelial cells. In the acidic environment of endosomes, FcRn binds albumin, thereby rescuing it from lysosomal degradation. Albumin is then returned to the cell surface and released into the circulation, significantly reducing its catabolic rate. Clinical evidence supports this mechanism: individuals with congenital loss of FcRn function, such as those with β2-microglobulin gene mutations, present with concurrent hypoalbuminemia and hypogammaglobulinemia. These findings underscore the indispensable role of FcRn in maintaining homeostasis of both IgG and albumin (9).

#### 2.4.3 Transport function

FcRn was initially recognized for its critical role in maternal-to-fetal IgG transport, a cornerstone of passive immunity. In the human placenta, FcRn is highly expressed in syncytiotrophoblasts, where it mediates transcytosis of maternal IgG across two cellular layers into the fetal circulation. This placental transport reaches peak efficiency during late gestation, with the majority of fetal IgG accumulation occurring in the final weeks of pregnancy. In fact, term neonates often exhibit serum IgG levels 20–30% higher than those of their mothers, highlighting the efficiency of FcRn-mediated transfer and its role in conferring passive immunity at birth (10). Beyond the placenta, FcRn also mediates transcellular IgG transport across other epithelial barriers. In neonatal rodents, for instance, FcRn facilitates the uptake of maternal IgG from ingested milk in the intestine (11). In this process, FcRn binds IgG at the acidic luminal surface, internalizes the complex, and releases IgG at the basolateral side under neutral pH, allowing its entry into the bloodstream. Although IgA is the predominant immunoglobulin in adult human mucosa, there is evidence that FcRn also mediates IgG transport at mucosal interfaces such as the respiratory and gastrointestinal tracts. Here, FcRn may deliver IgG from the circulation to the luminal surface to enhance mucosal immunity, or conversely, internalize luminal IgG or immune complexes for antigen sampling and clearance (12). Of note, certain pathogens exploit this FcRn-dependent transcytosis pathway: for example, HIV-1 has been shown to cross mucosal barriers by binding to IgG and engaging FcRn on genital tract epithelium (13).

#### 2.4.4 Other immunological functions

Recent studies have expanded our understanding of FcRn beyond its role in IgG and albumin recycling, highlighting its active participation in immune regulation. FcRn enhances neutrophil phagocytosis of IgG-opsonized pathogens by promoting more efficient phagosome formation, thereby augmenting microbial killing capacity (14). In glomerular podocytes, FcRn contributes to the clearance of immune complexes from the glomerular basement membrane, thus preventing immune complex–mediated tissue injury. Furthermore, FcRn is expressed in professional antigen-presenting cells such as dendritic cells, macrophages, and B lymphocytes. In these cells, FcRn facilitates the uptake and prolonged retention of IgG-bound antigens, enhancing their presentation to T cells (15). Through these mechanisms, FcRn plays a pivotal role in immune complex processing and regulation of the magnitude and duration of immune responses.

## 3 Overview of IgG biology

### 3.1 Structure and function of IgG antibodies

IgG is the most abundant immunoglobulin in human serum, comprising approximately 70-85% of total immunoglobulins. The IgG molecule consists of two heavy chains and two light chains, forming a “Y”-shaped structure: the upper arms are the Fab region, containing the variable regions responsible for specific antigen recognition, while the lower portion is the Fc region, a constant domain that mediates effector functions. IgG recognizes and neutralizes pathogens and toxins via its Fab region, while the Fc region links to innate immune effector mechanisms, such as the complement system and phagocytic cells. Human IgG is classified into four subclasses—IgG1, IgG2, IgG3, and IgG4—based on subtle differences in the structure of the heavy chain constant region. Despite a sequence homology of over 90% among the four subclasses, significant differences exist in the hinge region and the upper part of the CH2 domain (16). These regions determine the antibody’s ability to bind Fc receptors and complement. Overall, IgG1 and IgG3 possess long, flexible hinge regions, which facilitate the formation of antigen-antibody complexes and efficiently recruit complement and activate Fcγ receptors, making them the most potent in mediating effector functions such as phagocytosis, ADCC, and classical complement activation. In contrast, IgG2 and IgG4 have shorter or structurally distinct hinge regions, resulting in lower affinity for complement and Fcγ receptors: IgG2 typically activates complement or binds certain Fcγ receptors only when the antigen is densely clustered, while IgG4, due to a key residue mutation in the constant region, fails to fix complement and is inefficient at triggering Fc receptor pathways (17). Notably, IgG4 also exhibits a unique phenomenon known as “Fab arm exchange,” where two IgG4 molecules exchange one heavy and one light chain, producing functionally monovalent antibodies (each antibody molecule can bind two different antigens). This results in IgG4 displaying blocking rather than pro-inflammatory activity in certain contexts: IgG4 typically does not activate complement and competitively occupies antigen epitopes, potentially reducing the pathogenic effects of other antibodies or immune complexes. Overall, the structural and functional differences among IgG subclasses determine their intensity and mode of action in immune responses, and the combination of IgG subclasses in autoantibodies in autoimmune diseases can influence the disease’s pathogenesis and severity (**Table 1**).

**Table 1:** Overview of IgG subclass.

IgG subclass	IgG1	IgG2	IgG3	IgG4
Serum abundance(%)	60	32	4	4
Half-life (days)	21	21	7~21	21
Hinge region	Moderate	Short	Longest and most flexible	Moderate
FcγRI	+++	–	++++	++
FcγRIIA	+++	++	++++	++
FcγRIIB	+	–	++	+
FcγRIIC	+	–	++	+
FcγRIIIA	+++	+	++++	++
FcγRIIIB	+++	–	++++	–
FcRn binding	+++	+++	++	+++
C1q binding	++	+	+++	–
Function	Strong ADCC and CDC effects	Targeting polysaccharide antigens	Extremely strong ADCC and CDC effects, rapid response in the acute phase of infection	Does not activate complement and is involved in immunomodula-tion
Pathological antibody	Anti-AChR, anti-dsDNA, anti-AQP4, Anti-GPIIb/IIIa	Anti-β2-GPI, Anti-H2A	Anti-dsDNA, Anti-GBM	Anti-MuSK, Anti-PLA2R

ADCC, Antibody-dependent cell-mediated cytotoxicity; CDC, Complement-dependent cytotoxicity; AChR, acetylcholine receptor antibody; ds DNA, double-stranded DNA; AQP4, aquaporin 4; GP IIb/IIIa, glycoprotein IIb/IIIa; β2-GPI, beta-2 glycoprotein I; GBM, glomerular basement membrane; MuSK, muscle-specific kinase; PLA2R, M-type phospholipase A2 receptor.

"+" indicates weak binding affinity, "++" indicates moderate binding affinity, "+++" indicates strong binding affinity, "++++" indicates very strong binding affinity, and "-" indicates no binding affinity.

### 3.2 Mechanisms of IgG-mediated immune injury

#### 3.2.1 Complement activation

IgG antibodies activate the complement cascade through the classical pathway. When two or more IgG molecules aggregate on the surface of an antigen, their Fc regions bind to complement C1q, triggering the classical complement cascade (18). The activation products generated, such as C3a and C5a, are potent inflammatory mediators that attract neutrophils, monocytes, and other inflammatory cells to the site, leading to tissue infiltration and increased vascular permeability, thereby initiating an inflammatory response. Additionally, the complement cascade culminates in the formation of the membrane attack complex (C5b-9), which directly mediates cell lysis and tissue damage (19). Complement activation is particularly important in immune complex-mediated diseases.

#### 3.2.2 Fcγ receptor-mediated phagocytosis and cytotoxicity

The Fc region of IgG binds to Fcγ receptors (FcγRs) expressed on the surface of immune cells, triggering various cellular effector functions. Macrophages and neutrophils, upon binding IgG-opsonized target cells or immune complexes through high-affinity Fcγ receptors such as FcγRI, undergo phagocytosis and release proteolytic enzymes and reactive oxygen species (ROS), leading to tissue cell damage (20, 21). NK cells, upon binding IgG via FcγRIII, mediate antibody-dependent cellular cytotoxicity (ADCC) and kill target cells bearing self-antigens, such as autologous tissue cells (22). Additionally, the aggregation of FcγRs activates monocyte-macrophage cells to release pro-inflammatory cytokines (TNF-α, IL-1), amplifying the local inflammatory response (23). Under normal conditions, the body also expresses inhibitory Fcγ receptors (FcγRIIB) to balance these effects and prevent excessive inflammation. However, in autoimmune diseases, the presence of large amounts of IgG autoantibodies leads to persistent immune complex stimulation, with activating Fcγ receptor signals often dominating, surpassing the regulatory capacity of inhibitory signals, ultimately resulting in chronic inflammation and damage to self-tissues (24).

#### 3.2.3 Immune complex deposition

When self-antigens form soluble immune complexes with IgG antibodies, these complexes can deposit on vascular walls or tissues, triggering localized immune pathological damage. The deposited immune complexes continuously activate the complement cascade and release chemotactic factors, attracting inflammatory cells such as neutrophils to the deposition sites. Additionally, the IgG on the deposits can interact with FcγR-positive cells in the local tissue, such as tissue macrophages and dendritic cells, amplifying the inflammatory response (25).

## 4 IgG-mediated autoimmune diseases

Autoimmune diseases arise from an imbalance in immune tolerance, leading to the immune system erroneously attacking self-tissues. The pathogenesis of these diseases involves complex immune regulation disorders and effector loops. A key component in this process is the production and pathogenic role of autoantibodies, primarily of the IgG class. Extensive research has demonstrated that IgG autoantibodies play a central role in the pathogenesis of various autoimmune diseases, resulting in multi-organ inflammation and tissue damage. Given the pivotal pathogenichase of IgG in autoimmune diseases, effective strategies for regulating and clearing pathogenic IgG have become an important therapeutic approach. Rituximab depletion of B cells has been reported to improve diseases such as SLE, RA, Sjogren’s syndrome, AAV, and IgG4-RD. Recent clinical studies have highlighted the potential of FcRn inhibitors, which accelerate the clearance of circulating IgG and reduce pathogenic autoantibody levels, showing promise in delaying disease progression (26–28).

### 4.1 Systemic lupus erythematosus

SLE is characterized by the production of multiple autoantibodies, the vast majority of which belong to the IgG subclass (anti-double stranded DNA (dsDNA), anti-Smith antigen (Sm), and other nuclear antibodies). These IgG autoantibodies form immune complexes that deposit in various tissues and organs throughout the body, particularly in the glomerular basement membrane, which serves as the immunological basis for lupus nephritis (29). The deposition of circulating immune complexes can also affect the skin, joints, blood vessels, and other areas, causing corresponding clinical manifestations such as lupus rashes, arthritis, and vasculitis. Since IgG-mediated immune complexes are central to the pathogenesis of SLE, it is considered a classic immune complex-mediated disease. Characteristic evidence includes granular deposition of immunoglobulins and complement in renal biopsies, and complement consumption with hypocomplementemia observed during active disease phases (30). The levels of IgG autoantibodies are often correlated with disease activity, for instance, elevated anti-dsDNA titers frequently indicate disease flare-ups or worsening of nephritis. Therefore, removing or reducing pathogenic IgG antibodies (through high-dose intravenous immunoglobulin replacement or FcRn inhibitors to lower IgG levels) is considered a promising therapeutic strategy to mitigate immune complex-mediated tissue damage.

### 4.2 Rheumatoid arthritis

RA is another typical IgG-mediated autoimmune disease. In RA patients, autoantibodies such as rheumatoid factor (RF) and anti-citrullinated peptide antibodies (ACPA) are present, with ACPA primarily being pathogenic IgG subclasses. ACPA forms immune complexes with self-antigens that deposit in the synovial tissues of the joints and can bind with RF (an autoantibody targeting the Fc region of IgG) to form larger complexes, amplifying the pro-inflammatory effects of the immune complexes (31). Within the synovial membrane, IgG immune complexes activate resident macrophages and dendritic cells via Fcγ receptors, prompting them to secrete large amounts of pro-inflammatory cytokines (TNF-α, IL-6), leading to synovitis and cartilage and bone erosion. Meanwhile, complement activation through immune complexes produces chemotactic factors like C5a, which attract neutrophils into the synovial fluid. The proteases and reactive oxygen species released by these cells further exacerbate joint structural damage (32). In summary, IgG-mediated immune complexes and Fc receptor pathways play a central role in the inflammatory processes and tissue damage in RA.

### 4.3 ANCA-associated vasculitis

AAV are characterized by pathogenic IgG antibodies against neutrophil cytoplasmic antigens (mainly myeloperoxidase MPO or proteinase 3 PR3). Unlike the immune complex deposition mechanism seen in SLE and RA, in AAV, IgG autoantibodies mediate tissue damage primarily by directly activating circulating neutrophils. Under inflammatory stimuli, neutrophils expose MPO/PR3 antigenic epitopes on their surface, and ANCA IgG binds these antigens and interacts with Fcγ receptors on the neutrophil surface, causing excessive neutrophil activation. Activated neutrophils undergo respiratory burst and degranulation, releasing large amounts of reactive oxygen species and proteolytic enzymes that directly attack the vascular endothelium and surrounding tissues, resulting in necrotizing small vessel vasculitis and tissue damage (33). In the glomeruli and alveolar capillaries, ANCA-mediated neutrophil overactivation can cause rapidly progressive necrotizing glomerulonephritis and pulmonary hemorrhage (34). A study has clarified the important role of the complement system: ANCA-induced neutrophil activation can activate complement via the alternative pathway, releasing large amounts of C5a, which further recruits and activates more neutrophils. This positive feedback loop amplifies the inflammatory response and is considered a key mechanism in the ongoing damage seen in AAV. Drugs that block C5a receptors have shown clinical efficacy in reducing neutrophil recruitment and alleviating vascular inflammation (34). Strategies to directly reduce pathogenic IgG levels (using FcRn inhibitors to decrease ANCA concentrations) hold promise as a new direction in the treatment of AAV, providing a steroid-free therapeutic approach for patients.

### 4.4 Sjögren’s syndrome

Sjögren’s syndrome is an autoimmune disease primarily affecting the exocrine glands (such as lacrimal and salivary glands), leading to dry eyes and mouth, and is a classic example of a disease mediated by both humoral and cellular immunity. Characteristic autoantibodies include anti-Sjögren’s Syndrome-Related Antigen A/Ro (SSA/Ro) and anti-Sjögren’s Syndrome-Related Antigen B/La (SSB/La) antibodies, which target ribonucleoprotein complexes and are of the IgG class. Anti-SSA/Ro and anti-SSB/La antibodies form immune complexes with their respective antigens, which are then taken up by plasmacytoid dendritic cells. Through intracellular Toll-like receptors (TLR7/9), these complexes are recognized, triggering the production of type I interferons and pro-inflammatory cytokines (35). Histologically, the tissues of Sjögren’s syndrome show significant infiltration of T and B lymphocytes, with germinal center-like structures forming in the salivary glands, indicating the importance of cellular immunity in driving chronic inflammation (36). However, IgG antibodies produced by B cells and plasma cells remain key effectors in the disease.

### 4.5 Dermatomyositis and polymyositis

Dermatomyositis (DM) and polymyositis (PM) are two typical inflammatory myopathies with some differences in their pathological mechanisms: DM is characterized by both humoral immunity-mediated vascular damage and T-cell-mediated muscle fiber injury, while PM predominantly involves T-cell-mediated muscle fiber damage. IgG autoantibodies play an important pathogenic role in DM but are less significant in PM. Some IgG antibodies in DM patients may recognize antigens on muscle microvascular endothelial cells or structures around capillaries. Upon binding these target antigens, these antibodies recruit complement cascades, ultimately leading to the formation of MACs that attack the endothelial cells, causing capillary destruction, impaired perfusion, and ischemic injury to muscle fibers (37). Specific autoantibodies associated with DM, such as anti-Jo-1, anti-Mi-2, anti-Transcription Intermediary Factor 1 (TIF1)-γ, and anti-Melanoma Differentiation-Associated Protein 5 (MDA5) antibodies, are mainly IgG antibodies. In anti-Jo-1 positive DM patients, IgG anti-Jo-1 binds to muscle microvascular endothelial cells and induces complement-dependent cytotoxic effects, leading to endothelial cell apoptosis (38). Intravenous immunoglobulin has proven to be effective in treating DM, and the use of FcRn inhibitors to lower IgG levels may offer a new therapeutic approach.

### 4.6 Antiphospholipid syndrome

Antiphospholipid syndrome (APS) is an autoimmune disease characterized by coagulation dysfunction mediated by IgG antiphospholipid antibodies. These autoantibodies, known as antiphospholipid antibodies (aPL), target phospholipid-binding proteins, with the primary antigens being β2-glycoprotein I (β2-GPI) and cardiolipin. The pathogenic aPL are mostly of the IgG subclass, with IgG1 and IgG2 being the most prevalent. IgG antibodies against β2-GPI can form immune complexes on the surface of vascular endothelial cells or platelets, leading to excessive expression of tissue factors and adhesion molecules on these cells. This activates the extrinsic coagulation pathway and platelets, promoting thrombosis (39). After binding the target antigens, IgG anti-β2-GPI/cardiolipin antibodies can recruit and activate the complement cascade, and the deposition of C5b-9 membrane attack complexes on endothelial cells and placental villi leads to local inflammation and cell damage, which may result in placental insufficiency and miscarriage. Since APS is primarily mediated by IgG antibodies, FcRn inhibitors that enhance IgG clearance rates present an attractive therapeutic approach.

### 4.7 IgG4-related disease

IgG4-related disease (IgG4-RD) is a chronic autoimmune inflammatory disease characterized by elevated levels of IgG4 subclass antibodies and IgG4-positive plasma cell infiltration in affected tissues. Patients often have multi-organ involvement, with lesions presenting as tumor-like masses or painless enlargement, accompanied by progressive fibrosis and organ dysfunction (40). Serum IgG4 levels are significantly elevated in most patients, with levels greater than 1.35 mg/dL being considered diagnostic (41). Although IgG4-RD is marked by abnormalities in IgG4, it differs from typical IgG-mediated organ-specific autoimmune diseases in that no unified pathogenic target antigen has been identified. Some studies suggest that certain self-antigens may be associated with IgG4-RD: IgG4 autoantibodies against galectin-3 have been detected in some patients, and in IgG4-related pancreatitis, autoantibodies against pancreatic proenzyme have also been identified (41, 42). IgG4 typically does not activate the classical complement cascade. This property results in tissue lesions in IgG4-RD often lacking significant neutrophil infiltration or complement-mediated damage (43). Instead, IgG4-related lesions typically feature lymphoplasmacytic infiltration and fibrosis, suggesting that IgG4 may mediate disease by depositing immune complexes and inducing chronic fibrotic inflammation. This suggests that IgG4 antibodies can mediate tissue damage under certain circumstances, albeit via different mechanisms compared to IgG1 and other subclasses. Theoretically, FcRn blockade could reduce circulating IgG4 levels and potentially alleviate the disease. Existing studies have shown that, in addition to antibodies, the immune mechanism of IgG4-RD also includes T cell subsets, cytokines and fibrotic pathways (44). Therefore, further verification of the efficacy of FcRn inhibitors is still needed.

## 5 FcRn inhibitors

In systemic diseases such as rheumatic immunologic disorders, IgG autoantibodies are central pathological factors driving the disease. The different subclasses and modes of action of IgG antibodies collectively determine the disease phenotype and target organ damage. FcRn inhibitors are designed to block the interaction between FcRn and IgG, thereby accelerating IgG catabolism and reducing its circulating levels. Compared to broad-spectrum immunosuppressive treatments, FcRn inhibitors selectively lower IgG levels without affecting other immune components, resulting in fewer side effects. Moreover, FcRn inhibitors indirectly target antibodies, terminating their effects, and may be particularly effective in treating certain refractory autoimmune diseases. In summary, FcRn inhibitors have the potential to become a new therapeutic target in the field of autoimmune diseases.

**Figure 1:**
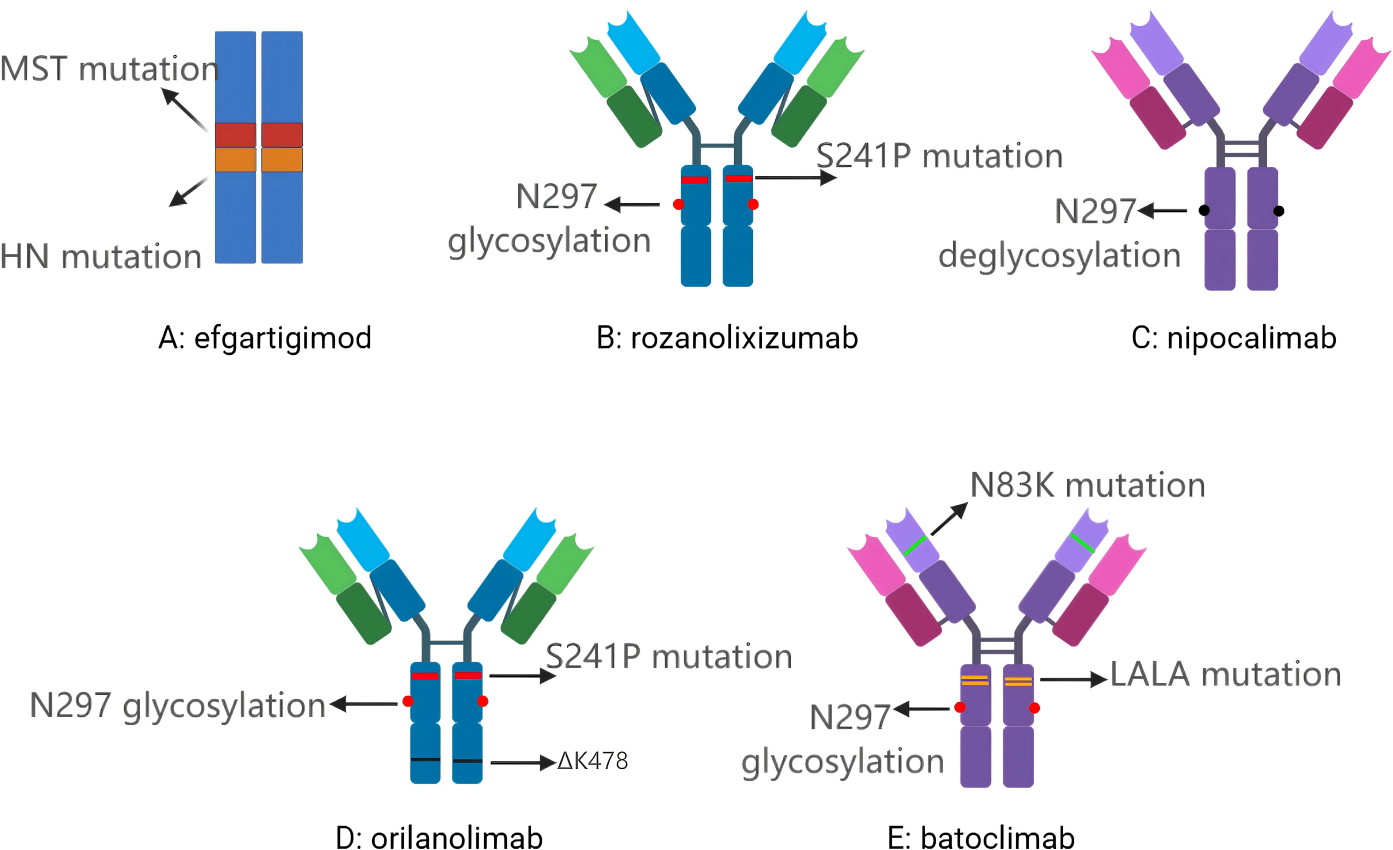
**(A)** Efgartigimod, a humanized IgG Fc fragment derivative; **(B)** Rozanolixizumab, a humanised IgG4 anti-FcRn monoclonal antibody; **(C)** Nipocalimab, a fully human IgG1 anti-FcRn monoclonal antibody; **(D)** Orilanolimab, a humanised IgG4 anti-FcRn monoclonal antibody; **(E)** Batoclimab, a human IgG1 anti-FcRn monoclonal antibody. MST mutation: Includes M252Y/S254T/T256E, altering the local amino acid composition to allow FcRn to bind Fc more strongly, thus extending the recovery half-life of IgG in cells; HN mutation: Includes H433K/N434F, which together with the MST mutation make up the ABDEG technology; N297 glycosylation: The asparagine residue at position 297 on the structural domain of IgG Fc CH2 is the natural glycosylation site ; S241P mutation: Serine at position 241 was mutated to proline with the aim of introducing rigidity and preventing Fab-arm exchange; N297 deglycosylation: Elimination of ADCC and CDC effects by de-glycosylation, thereby switching off FcγR/C1q binding; ΔK478: Lysine deletion at position 478 facilitates antibody conformational consistency and quality control; N83K mutation: Mutation of asparagine to lysine at position 83 of the heavy chain variable region for structural stability; LALA mutation: Leucine at positions 234 and 235 were both mutated to alanine, which significantly weakened the binding ability of FcγR and C1q and inhibited ADCC and CDC.

FcRn inhibitors are a novel class of therapeutics that target the FcRn-IgG interaction to reduce the levels of circulating pathogenic IgG, thus providing therapeutic benefits in IgG-mediated autoimmune diseases. These inhibitors come in various forms, including Fc fragment derivatives and anti-FcRn monoclonal antibodies. Several drugs have already been approved for clinical use or are currently undergoing clinical research. A representative drug of Fc fragment derivatives is efgartigimod, while representative FcRn monoclonal antibodies include rozanolixizumab, nipocalimab, orilanolimab, and batoclimab (**Table 2**, **Figure 1**).

**Table 2:** Overview of different types of FcRn inhibitors.

FcRn inhibitors	Efgartigimod (ARGX-113)	Rozanolixizumab (UCB7665)	Nipocalimab (M281)	Orilanolimab (SYNT001) (ALXN1830)	Batoclimab (IMVT-1401) (RVT-1401) (HBM9161)	IMVT-1402
Structure	Engineered IgG1-Fc fragment carrying ABDEG mutations.	Humanised IgG4 anti-FcRn monoclonal antibody with S241P mutation.	Fully human deglycosylated IgG1 anti-FcRn monoclonal. antibody	Humanised IgG4 anti-FcRn monoclonal antibody with S241P mutation.	Fully human IgG1 anti-FcRn monoclonal antibody.	Similar to batoclimab.
Mechanistic features	High affinity inhibitor of FcRn, competitive inhibition under acidic and neutral pH.	Targets the IgG-binding region of FcRn with high affinity.	Targets the IgG-binding region of FcRn with high affinity; minimal interference with albumin.	Targets the IgG-binding region of FcRn with high affinity.	Targets the IgG-binding region of FcRn with high affinity; more pronounced effect on serum albumin.	Targets the IgG-binding region of FcRn with high affinity; avoid blocking the albumin binding domain.
Clinical trials	Several phase III trials have been completed.	Several phase III trials have been completed.	Several phase III trials have been completed.	Several phase I trials have been completed. Development has now been terminated.	Phase-III gMG met primary end-point; company focus on next-gen IMVT-1402.	Several Phase I clinical studies are underway.
Approval status	Approved by FDA (IV, 2021; SC, 2023); also approved in Japan, EU, UK, China, etc.	Approved by FDA (2023); also approved in Japan, EU, UK.	Approved by FDA(2025)	Unapproved.	Unapproved.	Unapproved.
Indications	gMG (anti-AChR+ adults); phaseIII in CIDP, ITP, PV, BP, NMOSD.	gMG (anti-AChR+/MuSK+); phase-III in CIDP, MOGAD, SLE.	gMG (anti-AChR+/MuSK+); phase-III in wAIHA, RA, Sjögren, HDFN.	Formerly explored in PV, gMG, ITP, wAIHA.	gMG, CIDP, Graves’ ophthalmopathy, ITP.	gMG, RA, CLE, Sjögren’s syndrome
Adverse events	Headache, infection, injection site reactions	Headache, diarrhoea, injection site reactions	Infection, headache, peripheral edema, hyperlipidemia; adverse events are comparable to placebo.	Only mild headache and chills were reported in early trials.	Decrease in serum albumin; increase in LDL-C; headache and infections are less frequent.	Less data; no effect on albumin and LDL-C.
NCT Number	NCT03457649 (45), NCT03669588 (26), NCT04188379 (46)	NCT02220153 (47), NCT03971422 (27), NCT02718716 (48)	NCT02828046 (49), NCT04991753 (50), NCT04951622 (51), NCT03842189 (52)	NCT03643627 (53), NCT03075904 (54)	NCT03971916 (55), NCT04346888 (56), NCT03922321 (57), NCT05403541	NCT06980805, NCT06979531, NCT06754462, NCT07039916

gMG, Generalised myasthenia gravis; AChR, Acetylcholine receptor; CIDP, Chronic inflammatory demyelinating polyneuropathy; ITP, Immune thrombocytopenia; PV, Pemphigus vulgaris; BP, Bullous pemphigoid; NMOSD, Neuromyelitis optica spectrum disorder; MuSK, Muscle-specific kinase; MOGAD, Myelin-oligodendrocyte glycoprotein antibody disease; SLE, Systemic lupus erythematosus; wAIHA, Warm autoimmune hemolytic anemia; RA, Rheumatoid arthritis; HDFN, Haemolytic disease of the fetus and newborn; LDL-C, Low density lipoprotein cholesterol; SC, Subcutaneous; IV, Intravenous administration; CLE, Cutaneous lupus erythematosus.

### 5.1 Efgartigimod (ARGX-113)

Efgartigimod is a humanized IgG Fc fragment derivative. It leverages ABDEG technology to enhance its affinity for FcRn, which enables it to bind with FcRn at both acidic and physiological pH levels (45). The concept of ABDEG technology was first introduced in a study that engineered the human IgG Fc region to increase its affinity for FcRn with reduced pH dependence, thereby competitively blocking endogenous IgG recycling and promoting its degradation *in vivo (* 58). Its mechanism of action involves binding to FcRn, thereby blocking its interaction with IgG, which results in decreased serum IgG levels. Pharmacokinetic studies indicated that at a dosage of 50 mg/kg, efgartigimod exhibited a volume of distribution of 15–20 L, a half-life of 80–120 hours, and a bioavailability of approximately 50% (59). A first-in-human study involving 62 healthy volunteers indicated that a single administration of efgartigimod reduced IgG levels to 50% of baseline, while multiple doses further reduced IgG levels to an average of 75% of baseline. Approximately 8 weeks after the final dose, IgG levels returned to baseline. The reductions in IgG2 and IgG3 were similar to those of IgG1, while the reduction in IgG4 was less pronounced. Furthermore, no significant decreases were observed in the serum levels of IgA, IgD, IgE, IgM, or albumin (NCT03457649).

Currently, there are numerous clinical studies investigating the use of efgartigimod for treating Myasthenia Gravis (MG), Immune Thrombocytopenic Purpura, Chronic Inflammatory Demyelinating Polyneuropathy, and Pemphigus Vulgaris. A Phase 3 clinical trial involving 167 patients with Myasthenia Gravis indicated that more of those in the efgartigimod group were Myasthenia Gravis Activities of Daily Living Scale (MG-ADL) responders in cycle 1 than in the placebo group, with an odds ratio of 4.95 (95% CI: 2.21-11.53, p<0·0001) (NCT03669588) (26). A Phase 3 clinical trial involving 131 patients with Immune Thrombocytopenic Purpura indicated that 22% of patients receiving efgartigimod reached the primary endpoint compared with 5% of those receiving placebo(NCT04188379) (46). Multiple Phase II and Phase III clinical trials are still underway.

### 5.2 Rozanolixizumab (UCB7665)

Rozanolixizumab is a humanised IgG4 anti-FcRn monoclonal antibody that targets the IgG-binding region of FcRn, preventing IgG binding to FcRn (60). A Phase 1 clinical trial involving 49 healthy volunteers indicated that the serum total IgG concentration decreased in a dose-dependent manner after intravenous and subcutaneous administration of rozanolixizumab. Rozanolixizumab significantly reduced serum IgG levels by up to 57% in a dose-dependent manner in healthy subjects, with effects peaking 4–10 days after administration. At the highest dose, the average decrease in IgG typically reached its maximum between days 7 and 10, and IgG generally returned to baseline by day 57. Rozanolixizumab exhibited dose-dependent pharmacokinetics, with increasing systemic exposure and peak concentrations observed across intravenous doses. Peak levels were reached rapidly following Intravenous (IV) administration, while subcutaneous dosing led to slower absorption and delayed time to maximum concentration. There were no deaths or serious adverse events reported during the study (NCT02220153) (47).

In a phase 3 clinical trial involving 200 Myasthenia Gravis patients, researchers assessed the efficacy of rozanolixizumab for Myasthenia Gravis by using the MG-ADL, Myasthenia Gravis Composite Score (MGC), Quantitative Myasthenia Gravis Score (QMG), and MG symptoms Patient-Reported Outcome (PRO) scores. The trial indicated that rozanolixizumab demonstrated significant clinical and statistical improvements compared to the placebo control group (NCT03971422) (27). In a phase 2 clinical trial in ITP, results showed that the percent reductions in IgG levels from baseline were 60.0% and 52.3% in the 20- and 15-mg/kg single-dose cohorts, respectively, whereas the reductions were 63.8%, 49.9%, and 43.6% in the 10-, 7-, and 4-mg/kg multiple-dose cohorts, respectively (NCT02718716) (48). There were no significant changes in serum IgA, IgM and IgE levels. The study showed that a single subcutaneous dose of high-dose rozanolixizumab (15 or 20 mg/kg) led to a marked platelet increase by day 8, with over 50% of patients reaching ≥50 × 10^9^/L. In contrast, lower response rates and delayed platelet recovery were observed in the low-dose multiple-dose groups. Additionally, most patients with baseline bleeding symptoms experienced resolution by day 8. Overall, rozanolixizumab can significantly reduce serum IgG levels and improve patients’ symptoms.

### 5.3 Nipocalimab (M281)

Nipocalimab is a fully human IgG1 anti-FcRn monoclonal antibody that targets the IgG-binding region of FcRn, prevents IgG binding to FcRn. Through deglycosylation modifications, nipocalimab’s potential to trigger inflammatory responses was significantly reduced (61). This approach builds upon foundational research in Fc glycoengineering that has profoundly influenced the design of therapeutic antibodies (62). A phase 1 clinical trial involving 50 healthy volunteers indicated that intravenous infusion of single ascending doses up to 60 mg/kg induced dose-dependent serum IgG reductions, which were similar across all IgG subclasses. Multiple weekly doses of 15 or 30 mg/kg achieved mean IgG reductions of ≈85% from baseline and maintained IgG reductions ≥75% from baseline for up to 24 days. In this trial, nipocalimab showed dose-dependent pharmacokinetics, with serum concentrations increasing proportionally with dose, a consistent time to maximum serum concentration (Tmax) of 2 hours, a dose-dependent prolongation of terminal half-life (t_1_/_2_;), and a decrease in overall clearance (CL) at higher doses. Here, nipocalimab administration was well tolerated in all cohorts evaluated (NCT02828046) (49).

In a phase 2a clinical trial, investigators evaluated the therapeutic efficacy of nipocalimab in seropositive rheumatoid arthritis by designating the change from baseline in the Disease Activity Score 28 using C-reactive protein (DAS28-CRP) at Week 12 as the primary endpoint. Secondary endpoints included American College of Rheumatology (ACR) response rates (ACR20/50/70/90), DAS28-CRP remission and low disease activity rates, Clinical Disease Activity Index (CDAI), Health Assessment Questionnaire-Disability Index (HAQ-DI), and multiple patient-reported outcomes (PROs) such as joint pain severity and fatigue scores. In this trial, the primary endpoint did not reach statistical significance. Improvements were observed across all secondary endpoints, with higher ACR response rates, greater reductions in CDAI scores, and enhanced PROs. Pharmacodynamically, nipocalimab induced a rapid, significant, and reversible reduction in total IgG, anticitrullinated protein antibody (ACPA) IgG, and circulating immune complexes, while leaving inflammatory markers such as CRP unchanged. Notably, clinical improvement correlated with ACPA reduction, particularly in participants with higher baseline ACPA levels (NCT04991753) (50). Although the primary endpoint (DAS28-CRP) in this study did not reach statistical significance, nipocalimab treatment still demonstrated a consistent trend of clinical improvement, suggesting its potential in the treatment of rheumatoid arthritis. In addition, nipocalimab significantly and reversibly reduced serum total IgG, ACPA, and circulating immune complexes, confirming that its mechanism of action successfully achieved “target engagement.” The sample size and treatment duration of this trial may have influenced the outcomes. Moreover, the study population consisted of RA patients who were unresponsive to TNF-α antagonists, raising the question of whether this represents a distinct clinical subtype of RA and whether such a subtype may affect changes in DAS28-CRP. Further investigation is warranted to address these possibilities.

In the Phase II UNITY study, investigators administered weekly intravenous infusions of nipocalimab to pregnancies at high risk of early-onset severe haemolytic disease of the fetus and newborn (HDFN), and the results showed that 54 % (7/13) of pregnancies reached ≥32 weeks of gestation and achieved live birth without the need for intrauterine transfusion, markedly superior to historical high-risk cohorts. The UNITY data provided the first validation of the potential translational value of FcRn blockade in preventing maternal–fetal IgG transfer and delaying or avoiding severe fetal anemia. No hypoalbuminemia, peripheral edema, or hyperlipidemia was observed in this trial. The study also explored dosing in pregnant women and fetuses to support the clinical implementation of FcRn inhibitors during pregnancy(NCT03842189) (52). In the Vivacity−MG3 phase III randomized controlled trial, bi−weekly administration of nipocalimab at 15 mg/kg reduced median serum IgG by about 75 % as early as week 2 and maintained roughly a 69 % reduction through week 24. Overall safety was acceptable, but it is noteworthy that peripheral edema occurred in 11 % of patients, and those who developed edema reportedly had normal albumin levels. Laboratory tests showed increases in total cholesterol, HDL, and LDL, yet no short−term rise in cardiovascular risk was observed (NCT04951622) (51).

### 5.4 Orilanolimab (SYNT001) (ALXN1830)

Orilanolimab is a humanised IgG4 anti-FcRn monoclonal antibody that disrupts the binding of FcRn and IgG. A phase 1a clinical trial involving 200 suggested that orilanolimab showed nonlinear pharmacokinetics, with Cmax and areas under the curve (AUC) increasing disproportionately at higher doses. The half-life increased with dose (0.64 to 7.79 hours), and clearance decreased, indicating target-mediated disposition. The distribution volume remained near plasma volume, suggesting limited distribution beyond blood. In addition, data from this study indicated that besides lowering serum IgG levels, orilanolimab also reduced immune complexes and had a modulatory effect on the inflammatory response caused by immune complexes (NCT03643627) (53).

A phase 1b/2 clinical trial involving 8 pemphigus patients indicated that weekly intravenous orilanolimab (10 mg kg^-^¹ for five weeks) swiftly reduced total serum IgG by nearly 60 % and markedly lowered circulating IgG–immune complexes. 75 % of participants showed early and sustained improvement in Pemphigus Disease Area Index (PDAI) and lesion healing. Concordantly, pathogenic anti−desmoglein 1/3 autoantibody titres fell in most responders, supporting FcRn blockade as a promising strategy for rapid disease control in pemphigus. Orilanolimab was well-tolerated, with headache as the most common adverse event(NCT03075904) (54). AstraZeneca has announced the cessation of further research and development of the drug. This decision was mainly driven by AstraZeneca’s commercial considerations rather than scientific inevitability.

### 5.5 Batoclimab (IMVT-1401) (RVT-1401) (HBM9161)

Batoclimab is a human IgG1 anti-FcRn monoclonal antibody that reduces serum IgG levels by preventing the binding of IgG to FcRn. A phase 1 clinical trial indicated that subcutaneous batoclimab displayed non−linear, target−mediated pharmacokinetics in healthy Chinese adults. The AUC and the peak plasma concentration (Cmax) increased in a more than dose-proportional manner at the dose examined. Pharmacodynamically, a single injection produced rapid, dose−dependent and reversible reductions in circulating total IgG, reaching mean nadirs of –21.0 % (340 mg), –39.8 % (510 mg) and –41.2 % (680 mg) on Day 11 before gradually returning to baseline during the 85−day follow−up. The treatment was well tolerated, with only mild adverse events, mainly influenza-like symptoms and rash(NCT03971916) (55).

A phase 2 clinical trial in Myasthenia Gravis indicated that all the batoclimab groups showed clinical improvement compared to the placebo group. The greatest reductions of serum total IgG levels were measured on day 43, 57% and 74% in the batoclimab (340 mg) and batoclimab (680 mg) groups, respectively, compared with 2% in the placebo group. The most common adverse reactions were hypercholesterolemia, hyponatremia, urinary tract infection, injection site reaction and peripheral edema, which were mostly mild. Laboratory indicators suggested a dose-dependent reversible decrease in serum albumin (maximum -32.9 %, restored 6 weeks after drug withdrawal), accompanied by a mild increase in cholesterol (peak approximately +28 %) (NCT04346888) (56). In a proof-of-concept (POC) and randomized, double-blind placebo-controlled trial, the trial was prematurely terminated due to a significant increase in serum cholesterol, which was dose-related. Common adverse reactions also included hypoalbuminemia and peripheral edema(NCT03922321) (57).

A phase 3 clinical study on Myasthenia Gravis achieved the primary endpoint of MG-ADL changes relative to baseline in the AChR+ population at 12 weeks. The results indicated that the high-dose group improved by 5.6 points (with an average reduction of 74% in IgG), while the low-dose group improved by 4.7 points (with an average reduction of 64% in IgG). Safety and tolerability were observed to be consistent with prior batoclimab studies (NCT05403541). The data are sourced from the official website of Immunovant—the company developing batoclimab. It should be noted that despite the favorable data from the Phase 3 clinical trial, Immunovant will not seek marketing approval for batoclimab but will shift its focus to the development of the second-generation version, IMVT-1402. This decision was mainly based on the observation in clinical trials that batoclimab could cause hypercholesterolemia and hypoalbuminemia.

### 5.6 IMVT-1402

Immunovant first announced the results from the phase 1 trial, including the single ascending dose (SAD) and the 300 mg subcutaneous (SC) multiple ascending dose (MAD) cohorts. In the SAD portion of the study, subcutaneously administered IMVT-1402 demonstrated a consistent reduction in IgG with potency that was similar to or greater than that of batoclimab. The safety data were generally favorable, with all adverse events (AEs) mild or moderate, and no significant reduction from baseline in serum albumin or increase in LDL-C observed at any timepoint measured. In the MAD portion, after four weekly 300 mg SC doses of IMVT-1402, the mean total IgG reduction from baseline in this MAD cohort was 63%, with no decrease in serum albumin below baseline and no increase in LDL-C above baseline observed. Treatment-emergent adverse events were observed to be mild or moderate in severity.

Subsequently, Immunovant announced the results from the 600 mg MAD cohort of the IMVT-1402. Four once-weekly SC injections of 600 mg IMVT-1402 reduced total IgG level by a mean of 74%, a potency that is similar to batoclimab at 680 mg that reduced IgG by 76%. A reduction of 80% was observed at steady state after about 6–8 weeks. Across all doses evaluated, treatments with IMVT-1402 were generally well tolerated with only mild or moderate treatment-emergent adverse events observed. Serum albumin and LDL-C at Day 29 (peak pharmacodynamic impact) did not show a significant decrease or increase, respectively, from baseline. These data are sourced from the official website of Immunovant.

IMVT-1402 exhibits IgG-lowering efficacy comparable to or greater than that of batoclimab, without inducing decreases in serum albumin or increases in LDL-C even at higher doses. As a result, it demonstrates a superior safety profile and greater developmental potential. IMVT-1402 is currently being investigated in clinical trials for diseases such as rheumatoid arthritis, Sjögren’s syndrome, cutaneous lupus erythematosus, and Myasthenia Gravis.

## 6 Personalized therapeutics

The individualized treatment with FcRn inhibitors holds significant clinical value. By tailoring the therapy based on various factors, such as the patient’s genetic background, cell subsets, surface markers, and drug metabolism differences, it is possible to optimize therapeutic efficacy, minimize adverse effects, and enhance both the precision and sustainability of treatment. Although personalized treatment faces certain challenges, with the advancement of precision medicine and technological innovations, it is expected to play an increasingly crucial role in the field of immunotherapy. This approach will offer patients more precise, safe, and effective treatment options.

### 6.1 The polymorphism of the FcRn

Research indicates that the polymorphism of the FcRn alpha chain gene (FCGRT), specifically the variable number tandem repeat (VNTR), influences the expression level of FCGRT and serum IgG concentrations (63). Homozygous individuals for VNTR3 tend to exhibit higher levels of FcRn expression, as well as stronger binding affinity to IgG (64). Additionally, studies have shown that patients who are homozygous for VNTR3 have a lower clearance rate of therapeutic monoclonal antibodies. These findings suggest that VNTR3 homozygous patients may be more suitable candidates for FcRn inhibitor therapy. Moreover, FcRn gene polymorphisms could potentially serve as a critical basis for drug dosage adjustment (64). Given that FcRn gene polymorphism affects IgG levels, it raises the question of whether it could offer a novel classification approach for autoimmune diseases, thereby identifying populations more likely to benefit from FcRn inhibitor treatment. However, one study has pointed out that stratification based on FcRn gene polymorphism does not show significant differences in the clinical characteristics or prognosis of patients with lupus nephritis (65). Therefore, further research is required to explore the relationship between FcRn gene polymorphisms and susceptibility to autoimmune diseases in greater depth.

### 6.2 Long live plasma cells

B cells play a crucial role in the pathogenesis of many autoimmune diseases. Long-lived plasma cells, by continuously secreting pathogenic autoantibodies, contribute to the chronic inflammatory processes observed in autoimmune diseases. These long-lived B cells, which do not undergo DNA synthesis and remain non-dividing, are characterized as longevity cells, while long-lived plasma cells do not express CD20 (66). As a result, conventional B cell-targeted therapies, such as cyclophosphamide and rituximab, are ineffective in depleting long-lived plasma cells. Therefore, long-lived plasma cells play a critical role in the pathogenesis of refractory autoimmune diseases, including treatment-resistant SLE and RA (67). FcRn inhibitors cannot directly deplete long-lived plasma cells. Instead, they can reduce the pathogenic antibodies produced by these cells. FcRn inhibitors may offer therapeutic benefits for patients with treatment-resistant autoimmune diseases driven by the persistent activation of long-lived plasma cells.

### 6.3 Cytokines

Certain abnormally elevated cytokines may serve as predictive biomarkers for therapeutic response to FcRn antagonists. The generation of antigen-specific plasma cells is dramatically decreased in IL-6−/− mice (68). In an experimental model of SLE, genetic deletion of IL-6 resulted in a reduction in the generation of pathogenic autoantibodies (69). A study indicated that B-cell Activating Factor (BAFF) can promote B cell survival and differentiation, and its levels are elevated in the serum of patients with MG (70). The increase in these cytokines is related to the overactivation state of B cells/plasma cells. In lupus patients, type-I interferon may drive the expansion of polyclonal plasma blasts, leading to hypergammaglobulinemia, especially elevated serum IgG levels (71). High levels of type-I interferon in serum may indicate a poorer response to B-cell depletion treatment or BAFF pathway blockade treatment in patients (72, 73), which suggests that these patients may benefit from FcRn inhibitors. In patients with rheumatoid arthritis, anti-citrullinated protein antibodies (ACPAs) can induce macrophages to secrete TNF-α (74), thereby contributing to the inflammatory cascade. Elevated serum TNF-α levels may therefore underscore the pathogenic role of autoreactive antibodies and highlight the therapeutic relevance of strategies aimed at reducing their production to attenuate TNF-α–mediated inflammation. Moreover, as previously noted, TNF-α has been shown to upregulate FcRn expression. Overall, the elevation of these cytokines may indicate that the patient is suffering from an autoimmune disease characterized by high titers of pathogenic antibodies. In such cases, the use of FcRn inhibitors to reduce antibody levels may help improve the patient’s prognosis. However, current research in this area is limited, and future studies will need to be designed with stratification based on the cytokine levels of the patient population to further elucidate the potential benefits of FcRn inhibition in specific subgroups.

### 6.4 Immune cell subsets

Alterations in some immune cell subsets may serve as predictors of the therapeutic effect of FcRn inhibitors. SLE patients who do not respond to belimumab treatment exhibit a higher pre-treatment proportion of memory B cells, and these non-responders also display elevated immunoglobulin expression levels within the memory B cell population. This observation suggests that FcRn inhibitors may be more suitable for patients with a predominance of memory B cells (75). Moreover, one study reported that a subset of SLE patients demonstrated an elevated ratio of plasmablasts to memory B cells (PB/M). These individuals were frequently associated with heightened disease activity and elevated total ANA-IgG levels. The study further noted that patients with a high PB/M ratio exhibited serological features indicative of B cell hyperactivity, including increased serum IgG levels and decreased IgM levels. These findings imply that enhancing IgG clearance through FcRn inhibition may offer clinical benefits for this patient subgroup (71). A study revealed significant alterations in dendritic cell (DC) subset proportions in the experimental autoimmune Myasthenia Gravis (EAMG) model, characterized by marked increases in conventional dendritic cell (cDC) 1 proportion, concomitant with a significant reduction in cDC2 proportion. Efgartigimod demonstrated notable efficacy in restoring homeostasis of DC subsets, as evidenced by normalization of cDC1 and cDC2 proportions. By rebalancing DC proportions, efgartigimod may effectively disrupt the vicious cycle of autoimmune activation in MG. These results suggest that FcRn inhibitors may confer greater therapeutic benefit in patients with an elevated cDC1/cDC2 ratio (76).

### 6.5 FcγRIIB

FcγRIIB is a negative regulatory receptor in the immune system that plays a crucial role in maintaining immune tolerance. By binding to immunoglobulins, it inhibits the activation of immune cells, thereby helping to reduce the accumulation of immune complexes and exerting significant immune regulatory effects. As the only FcγR expressed on B cells, FcγRIIB suppresses B cell activation upon binding with antigen-antibody complexes. One study observed a reduction in FcγRIIB expression in memory B cells from patients with SLE (77). Another study found that dendritic cells in patients with low disease activity in RA expressed higher levels of FcγRIIB compared to those with high disease activity (78). Given FcγRIIB’s role in negatively regulating immune responses, patients with low FcγRIIB expression or functional defects tend to exhibit hyperactive immune responses, especially in conditions where large quantities of immunoglobulins and immune complexes are produced. In such cases, FcRn inhibitors, by reducing the half-life of IgG and lowering IgG levels, can help mitigate the excessive activation of the immune response. Therefore, for patients with low FcγRIIB expression or those carrying dysfunctional FcγRIIB variants, the use of FcRn inhibitors may offer better therapeutic outcomes.

## 7 Combination therapies

### 7.1 Glucocorticoids

Glucocorticoids (GCs) significantly influence the production of IgG through multi-level immunoregulatory mechanisms. Their primary effects are mediated by binding to cytoplasmic glucocorticoid receptors, which translocate into the nucleus and modulate several key signaling pathways, including suppression of NF-κB, Activator Protein (AP)-1, and JAK-STAT pathways, thereby broadly inhibiting B cell activation, class switch recombination, and plasma cell differentiation (79). GCs downregulate the expression of essential transcription factors such as activation-induced cytidine deaminase, B Lymphocyte-Induced Maturation Protein 1 (Blimp-1), and X-box Binding Protein (XBP) 1, thus restricting IgM-to-IgG switching and antibody secretion. Moreover, GCs suppress T helper cells—particularly follicular helper T cells—by reducing the production of IL-4, IL-21, and CD40L, and inhibit the generation of B cell survival factors such as BAFF and IL-6 (80). Collectively, glucocorticoids inhibit humoral immune responses through both direct and indirect mechanisms, leading to a marked reduction in IgG levels, and are therefore widely used in the clinical management of antibody-mediated diseases. However, glucocorticoid treatment is often associated with numerous adverse effects. In the treatment of autoimmune diseases, a strategy to reduce glucocorticoid dosage by combining them with other immunosuppressive agents is sometimes employed, tailored to the individual patient’s condition. FcRn inhibitors, known for their capacity to reduce serum IgG levels, suggest the possibility of combining them with glucocorticoids for enhanced therapeutic effects. Currently, research on the combination of FcRn inhibitors and glucocorticoids remains limited. A case report indicated that efgartigimod combined with corticosteroid therapy can effectively manage a myasthenic crisis without the need for plasma exchange or Intravenous Immunoglobulin, and during maintenance treatment, it can alleviate symptoms, reduce antibody levels, and improve daily living activities, with minimal manifestation status maintained even after corticosteroid tapering (81). A clinical trial investigating the combination of efgartigimod with prednisone in the treatment of pemphigus has been completed. The research have shown that after combination therapy with efgartigimod, the dose of prednisone required to achieve complete remission was significantly lower than the conventional dosage (NCT03334058) (82). Although FcRn inhibitors primarily serve as adjunctive therapies in these studies, their introduction has enabled the maintenance of disease improvement while allowing for glucocorticoid dose reduction. This is particularly meaningful for minimizing glucocorticoid-associated adverse effects. Furthermore, a clinical trial evaluating the combination of intravenous methylprednisolone and efgartigimod in the treatment of neuromyelitis optica spectrum disorders is ongoing. Additional clinical trials are also in progress. Current studies focus on optimizing combination therapy regimens, including exploring strategies for glucocorticoid dose reduction.

### 7.2 Traditional immunosuppressive agents

Traditional immunosuppressive agents, such as methotrexate, cyclophosphamide, azathioprine, and cyclosporine, broadly affect T/B cell activation, cytokine secretion, and antibody generation. The pathogenesis of many autoimmune diseases often involves multiple targets and pathways, rendering therapies directed at a single target potentially less effective. The combination of FcRn inhibitors with traditional immunosuppressive drugs represents a multi-target intervention approach. FcRn inhibitors clear pre-existing pathogenic IgG, while traditional drugs suppress the generation of new antibodies, collectively blocking both upstream and downstream components of autoimmune responses. No studies have yet investigated such combination therapies. Patients with antibody-mediated autoimmune diseases, including but not limited to SLE, RA, Sjögren’s syndrome, and IgG4-related diseases, may benefit from this combined therapeutic approach.

### 7.3 Biological agents

Biological agents, as powerful tools in the treatment of autoimmune diseases, may also hold potential for synergistic effects when combined with FcRn inhibitors to enhance therapeutic outcomes. Telitacicept, a recombinant fusion protein that inhibits BAFF and A Proliferation-Inducing Ligan (APRIL), thus suppressing B cell maturation, has demonstrated significant therapeutic results in SLE. A clinical trial investigating the combination of efgartigimod and telitacicept in the treatment of Myasthenia Gravis is currently underway (NCT06827587). It is noteworthy that the trial design emphasizes a sequential therapy approach, with efgartigimod administered first, followed by telitacicept. This sequential strategy may be rooted in a logical progression: the FcRn inhibitor rapidly clears existing pathogenic antibodies to alleviate acute symptoms, followed by telitacicept to suppress the formation of new antibodies, thereby achieving long-term immune regulation. It should be noted that most biologics possess an Fc fragment and rely on FcRn to extend their half-life. Therefore, co-administration of FcRn inhibitors with certain biologics may in fact reduce the efficacy of the biologics due to a shortened half-life. The sequence and interval of administration between the two drugs can also influence the therapeutic outcome. However, this cannot deny the potential application value of the combined treatment of FcRn inhibitors and biological agents. A retrospective case study indicated that the combination of efgartigimod and telitacicept could alleviate the symptoms of gMG. The case under study was treated with telitacicept one week after the use of efgartigimod (83). Another retrospective real-world study suggested that telitacicept be used 2 to 4 weeks after the use of efgartigimod (84). Overall, in terms of treatment sequence, FcRn inhibitors were administered prior to biological agents. At present, the clinical evidence regarding the combined treatment of FcRn inhibitors and biologics remains limited. The clinical study (NCT06827587) mentioned above aims to explore the advantages and disadvantages of the combination of efgartigimod and telitacicept compared with monotherapy, as well as the optimal interval between administrations. A clinical trial evaluating the combination of nipocalimab and certolizumab (TNF-α inhibitor) for the treatment of RA has been completed (NCT06028438). Study results have not been submitted. Further evaluations of this approach will require additional clinical trial data.

## 8 FcRn inhibitors as alternatives to plasma exchange and intravenous immunoglobulin

FcRn inhibitors, plasma exchange (PLEX), and intravenous immunoglobulin (IVIg) represent key therapeutic modalities in the management of autoimmune diseases, yet they differ significantly in their mechanisms of action and clinical characteristics (85). FcRn inhibitors selectively block the FcRn, thereby accelerating the degradation of IgG antibodies without significantly affecting other immunoglobulin isotypes such as IgM or IgA. Their main advantages include high target specificity, prolonged efficacy, and non-invasiveness, making them particularly suitable for long-term management of chronic autoimmune disorders. However, FcRn inhibitors typically have a delayed onset of action, requiring several days to weeks, which limits their utility in acute crisis scenarios (85).

In contrast, PLEX rapidly removes circulating macromolecules—including various antibody subtypes, immune complexes, and complement components—by extracorporeal filtration. Its major strength lies in its rapid onset, often within hours, making it a first-line intervention in acute emergencies such as myasthenic crisis. Nonetheless, the clinical benefit is transient, necessitating repeated procedures over a short period. As an invasive therapy, PLEX is associated with a spectrum of potential complications, including allergic reactions, hemolysis, bleeding, hypocalcemia, infections, and catheter-related events (86). Moreover, its non-selective depletion of plasma proteins may exacerbate bleeding tendencies and increase the risk of immune dysregulation (86). FcRn inhibitors circumvent the need for plasma, offering potential pharmacoeconomic advantages for long-term use. IVIg provides broad immunomodulatory effects, including pathological antibody neutralization, saturating Fc receptors, promoting clearance of circulating immune complexes, with relatively rapid symptom relief. However, it is non-specific in action and carries risks such as infusion-related reactions, thromboembolic events, and renal impairment (87).

Both PLEX and IVIg rely on human blood products, posing a theoretical risk of infectious disease transmission. FcRn inhibitors, therefore, present a promising alternative for chronic maintenance therapy and may potentially replace PLEX and IVIg in selected patient populations. However, in acute, life-threatening scenarios, PLEX and IVIg remain indispensable. Additionally, FcRn inhibitors are ineffective in diseases not mediated by IgG, whereas PLEX and IVIg retain broader applicability across a range of autoimmune disorders.

## 9 Exploration of novel FcRn inhibitors

### 9.1 Engineered antibodies

The development of novel FcRn inhibitors mainly focuses on improving drug efficacy and optimizing safety. To enhance the therapeutic effect, researchers are dedicated to developing molecules with higher affinity to significantly improve FcRn inhibitiors and prolong the drug’s half-life in the body, thereby reducing dosing frequency. The impact of core fucose deletion on FcγRIIIa affinity was first systematically elucidated, laying the foundation for antibody glyco-engineering (88). Subsequent work developed the M252Y/S254T/T256E (YTE) and M428L/N434S (LS) Fc variants, both of which markedly enhance FcRn binding and extend antibody half-life, thereby representing one of the classic modifications for prolonging IgG circulation time and inaugurating systematic optimization of FcRn affinity (89, 90). Engineered antibodies are created by introducing specific amino acid mutations into the Fc fragment of IgG antibodies, which enhances their binding capacity to FcRn. For example, efgartigimod employs the MST-HN mutation strategy (45), significantly increasing its affinity for FcRn under different pH conditions. More efficient mutation strategies are still under investigation. Through engineering techniques, such as altering the glycosylation patterns of the Fc fragment, it is possible to significantly affect its interaction with other Fc receptors (FcγR), thereby reducing off-target effects and preventing interference with non-target molecules. A review synthesizes three complementary design routes: (i) host-cell pathway engineering (editing glycosyltransferases in Chinese Hamster Ovary (CHO) cell to control core fucosylation, terminal galactosylation, and sialylation), (ii) chemoenzymatic remodeling that removes native glycans with endoglycosidases and installs defined structures ex vivo, and (iii) sequence-level designs that ablate or reposition Fc glycosylation sites (N297A) to silence FcγR/C1q engagement. Functionally, afucosylation enhances FcγRIIIa binding and ADCC, increased galactosylation promotes C1q binding and CDC, whereas elevated sialylation or other Fc-silent variants—attenuate pro-inflammatory signaling while preserving antigen recognition. These principles underpin current strategies for configuring anti-inflammatory antibodies and for producing uniform glycoforms suitable for translation (91).

The humanized monoclonal antibodies can reduce immunogenicity and significantly lower the risk of immune responses triggered by the drug. In the early days, murine therapeutic monoclonal antibodies (mAbs) routinely elicited robust human anti-mouse antibody (HAMA) responses; consequently, investigators proposed the strategy of antibody humanization, in which only the antigen-binding complementarity-determining regions (CDRs) were retained and grafted onto human framework regions, thereby reducing xenogenicity at its source (92). In 1986, CDR grafting was first validated, and the concept was rapidly translated into the clinic, leading to development of the humanized antibody Campath-1H, which achieved lymphoma remission in 1988 (93). Subsequent structural studies revealed that the restoration of a small set of “Vernier” framework residues recovered the affinity lost during grafting, thus establishing framework back-mutation as a routine step in humanization workflows (94). Contemporary protocols combined deep-learning-based humanness scoring with T-cell epitope prediction tools, and thereby enabled computation-driven, low-immunogenicity design while markedly shortening experimental iteration cycles (95). Owing to these advances, more than half of the therapeutic antibodies approved to date have been humanized or fully human, and products that integrated CDR grafting with de-immunization approaches exhibited significantly lower anti-drug antibody incidence (96).

It should be noted that affinity is not the sole determinant in antibody design. A study highlighted that affinity-enhancing mutations often diminish framework thermodynamic stability or trigger nonspecific interactions and poor solubility. Conversely, compensatory stabilizing substitutions, framework charge re-engineering, N-glycosylation to shield hydrophobic hotspots, and integrated positive–negative selection can markedly improve developability without compromising affinity. The convergence of multi-objective computational protein design, high-throughput stress-based screening, and deep-sequencing-assisted data mining thus constitutes a new “co-optimization” paradigm in antibody engineering (97).

### 9.2 Small-molecule inhibitors and innovative drug-delivery modalities

Small molecule inhibitors typically exhibit better tissue penetration and bioavailability. The development of small molecule inhibitors that can efficiently and specifically bind to FcRn is a promising area of research. Affibody, a Swedish company, has developed ABY-039, a novel FcRn inhibitor that combines an FcRn binding domain and an albumin binding domain (98). This small molecule fusion protein improves the stability and safety of FcRn-targeting drugs by binding to albumin. Additionally, exploring alternative administration methods is a key research focus. For example, the design of orally administered FcRn inhibitors not only enhances patient compliance but also broadens the therapeutic range. It has been reported that both fetal and adult intestinal epithelial cells express FcRn and transport antibodies from the intestinal lumen to the bloodstream, providing a potential direction for the development of oral FcRn inhibitors (99). However, current FcRn inhibitors mainly consist of Fc fragment derivatives and monoclonal antibodies, which are limited by the first-pass effects and the degradation of stomach acid and digestive enzymes when taken orally. Oral FcRn inhibitors remain at the proof-of-concept stage. Future breakthroughs may come from the development of small molecules and the utilization of nanoparticle encapsulation technology (100). Through these optimization strategies, novel FcRn inhibitors hold the potential to provide more potent, safer, and more convenient therapeutic effects, offering significant benefits to patients with autoimmune diseases.

### 9.3 pH-dependent modulation strategies

The development of next-generation FcRn inhibitors based on new pharmacological mechanisms is a critical research direction for the future. Studies have shown that FcRn binds to IgG in an acidic environment but dissociates in a neutral environment, suggesting that the function of FcRn is pH-dependent (5). Based on this mechanism, researchers are continuously improving the affinity of FcRn inhibitors, enabling them to bind to FcRn in both acidic and neutral environments. It could be envisioned that future pharmacologic strategies targeting the endosomal pH gradient may disrupt the pH-dependent binding of IgG to FcRn, thereby modulating IgG recycling and homeostasis. Currently, several drugs have been reported to increase the pH of endosomes. Bafilomycin (101) and Concanamycin (102), as V-ATPase inhibitors, raise the pH of endosomes and are widely used in experimental settings for studying endosomes and lysosomes. However, their clinical use is limited due to their toxicity. Drugs such as chloroquine and hydroxychloroquine (103), commonly used to treat autoimmune diseases, have also been reported to elevate endosomal pH. However, their therapeutic effects in autoimmune diseases are not due to this action, but rather based on their significant anti-inflammatory properties.

### 9.4 Targeting endosomal–lysosomal trafficking pathways

The function of FcRn is highly dependent on the vesicular system, which is responsible for the transport and recycling of antibodies. This includes the transition from early to late endosomes, the recycling loop between endosomes and the cell membrane, and dynamic sorting processes involving lysosomes. Drugs that inhibit vesicular transport could potentially interfere with FcRn’s ability to recycle and transport IgG. Studies have shown that Rab GTPases are key regulatory molecules in vesicular transport, controlling the dynamic cycling of FcRn by promoting vesicle formation, transport, and fusion. Specifically, Rab11 is primarily involved in the recycling transport of FcRn from the endosome to the cell surface, while Rab7 plays a role in the transport process from late endosomes to lysosomes (104). Existing studies have shown that statins specifically inhibit Rab proteins (105). Developing specific inhibitors targeting these Rab proteins and their regulatory mechanisms could provide a novel strategy for interfering with FcRn function. It should be noted that these GTPases are also indispensable for other cellular programs. Rab11 governs recycling of MHC−I molecules that underlies antigen cross−presentation in dendritic cells (106). Broad Rab11 inhibition may therefore dampen adaptive immunity and tumour surveillance. Animal studies have demonstrated that inhibition of Rab7 may lead to pathological alterations such as degeneration of peripheral sensory neurons (107) and injury to podocytes (108). Although Rab protein inhibitors are non-selective and have associated side effects, this approach still holds exploratory value in future development. A study has shown that small molecule inhibition of Rab7 can inhibit the class awitching of B cells and the survival of plasma cells in lupus mice, thereby suppressing the autoantibody response (109). Inhibition of Rab proteins may exert a dual mechanism of action—by disrupting FcRn-mediated recycling and simultaneously limiting antibody production at the source.

### 9.5 FcRn degradation

Proteolysis Targeting Chimeras (PROTACs) are a novel class of small molecule drugs designed to induce the degradation of target proteins, rather than traditional functional inhibition, to achieve therapeutic effects (110). A PROTAC molecule consists of three main components: a target protein ligand, a linker, and an E3 ligase ligand. PROTACs work by simultaneously binding to the target protein and the E3 ligase, inducing the E3 ligase to ubiquitinate the target protein. This marks the target for recognition by the proteasome, leading to its degradation. Based on this proposed mechanism, it could be envisioned that PROTACs specifically targeting FcRn as the protein of interest may be designed in the future to promote its degradation. This strategy not only inhibits FcRn function but also reduces the overall expression level of the receptor, making it suitable for chronic diseases that require long-term suppression of FcRn function. At present, this method is still at the stage of theoretical speculation.

In summary, based on these proposed mechanisms, it could be envisioned that future drug development may be broadened through multiple innovative designs, potentially laying the groundwork for more efficient and precise therapeutic strategies. These approaches demonstrate great potential in addressing issues related to drug resistance and tolerance. However, these innovative concepts still require further refinement.

## 10 Discussion

Despite the promising therapeutic potential of FcRn inhibitors, several limitations and concerns remain in their clinical application. One of the major issues lies in the subclass-agnostic nature of FcRn blockade, which reduces all IgG subclasses indiscriminately. Inhibiting FcRn-mediated recycling leads to a global reduction in serum IgG levels, not only targeting pathogenic autoantibodies but also diminishing protective IgG required for immune defense. Clinical trials have frequently reported reductions in total IgG levels ranging from 50% to 80%. Such profound IgG depletion is comparable to iatrogenic hypogammaglobulinemia and may compromise humoral immunity, thereby increasing the risk of infections. This effect is similar to that observed with long-term intravenous immunoglobulin replacement or plasma exchange therapy. However, it is noteworthy that to date, clinical evidence has not shown a significant increase in the incidence of serious infections with FcRn inhibitors. This may be attributed to the reversible and controllable mechanism of action: the IgG-lowering effect is transient and serum levels typically return to baseline within months after treatment cessation (111). Furthermore, FcRn inhibition does not affect IgM or IgA levels, nor does it directly target B cells or T cells. Therefore, when administered in short courses, FcRn inhibitors generally exert a limited degree of immunosuppression. Nonetheless, for patients requiring long-term or repeated treatment cycles, sustained IgG depletion may pose cumulative risks. In particular, clearance of pathogen-specific antibodies may diminish immune memory derived from prior infections or vaccinations. It is thus recommended that patients undergo immunization status assessment before initiating FcRn antagonist therapy, with appropriate booster vaccinations administered when necessary. Live attenuated vaccines should be avoided during treatment to mitigate infection risk (112). While FcRn inhibitors differ mechanistically from traditional immunosuppressants and lack broad cytotoxic effects, long-term safety data are still limited. The impact of prolonged hypogammaglobulinemia on host defense, tumor immunosurveillance, and overall immune homeostasis remains unclear. Furthermore, the host may engage compensatory mechanisms in response to sustained IgG depletion, such as increased B-cell proliferation or augmented antibody synthesis, which warrant further investigation. Therefore, in the context of long-term maintenance therapy with FcRn inhibitors, clinicians and patients must carefully balance the potential risks of reduced IgG levels against disease activity. Individualized treatment plans, regular monitoring of immunoglobulin levels, and vigilance for infection or immune dysfunction are essential components of clinical management.

Although FcRn inhibitors have demonstrated favorable efficacy and safety profiles in adult populations, their use in special populations—including children, adolescents, pregnant, and lactating women—requires cautious consideration. The developing immune system in children is particularly reliant on IgG for defense against common pathogens; thus, marked IgG reduction may impair their protective immunity. To date, pediatric data remain sparse for most FcRn inhibitors, with the exception of nipocalimab, which has been studied in adolescents aged 12 and above with generalized Myasthenia Gravis (113). There is a lack of robust evidence to support FcRn inhibitor use in pediatric autoimmune disorders, underscoring the need for further clinical trials to evaluate age-appropriate dosing, safety, and potential developmental effects. Similarly, in women who are planning pregnancy or are currently pregnant, reducing maternal IgG may affect transplacental antibody transfer and compromise neonatal passive immunity. However, exceptions may exist in specific clinical scenarios: emerging studies suggest that FcRn blockade may offer fetal protection in cases of high-risk pregnancies complicated by alloimmune hemolytic disease (52). Thus, in the context of maternal–fetal medicine, FcRn inhibitors may serve as rescue therapy under strictly controlled conditions. In general, the application of FcRn inhibitors in special populations should follow a principle of caution and individualization. Unless there is clear, evidence-based benefit, long-term use in children and pregnant women should be avoided, and if deemed necessary, should be accompanied by enhanced clinical surveillance and supportive care.

Given the complex pathogenesis of autoimmune diseases, monotherapy is often insufficient to address all pathogenic mechanisms. Therefore, combining FcRn inhibitors with agents targeting complementary immunological pathways has emerged as a promising therapeutic strategy. B cell–depleting therapies such as rituximab can reduce the production of pathogenic autoantibodies, while FcRn antagonists eliminate circulating IgG autoantibodies. The combination of these approaches may achieve both “source reduction” and “downstream clearance,” resulting in a dual blockade of disease-driving antibodies. Similarly, in disorders where complement-mediated tissue injury plays a central role, complement inhibitors such as eculizumab can suppress antibody-triggered complement activation, whereas FcRn blockade reduces the overall burden of pathogenic IgG. Though mechanistically distinct, these agents may act synergistically, potentially yielding more profound clinical responses than either agent alone. Combining therapies that target multiple arms of the immune response may also improve efficacy, reduce corticosteroid dependency, and lower relapse rates. Currently, this area of research remains in its infancy. Rigorous clinical trials are needed to evaluate the safety, efficacy, and optimal timing of such combination regimens. Importantly, the potential benefits of combination therapy must be weighed against risks, including additive immunosuppression, increased infection susceptibility, and higher treatment costs. Therefore, careful patient selection and close monitoring are essential to balance therapeutic gain with safety.

Although FcRn has been widely recognized as a key regulator of IgG homeostasis, the notion that FcRn inhibition–induced IgG reduction is the principal mechanism underlying therapeutic efficacy remains subject to debate. A study showed that IVIg effectively ameliorated antiplatelet antibody–induced thrombocytopenia in FcRn-deficient mice, suggesting that the therapeutic effect of IVIg in acute murine ITP does not depend on FcRn expression (114). While one prevailing hypothesis posits that high-dose IVIg saturates FcRn through competitive binding, thereby accelerating the clearance of all IgG, including pathogenic antiplatelet antibodies (115), it is important to recognize that IVIg does not equate to selective FcRn blockade. Beyond IgG catabolism, IVIg exerts a broad spectrum of immunomodulatory effects—such as Fcγ receptor blockade and modulation of immune effector cell function—which complicates mechanistic interpretation. In contrast, FcRn-targeting monoclonal antibodies like rozanolixizumab exhibit high selectivity and operate via a clearly defined mechanism of action. Extrapolating findings from this murine model to human disease is further limited by several important considerations: the model represents an acute, passive transfer system lacking ongoing autoantibody production; murine FcγR and FcRn differ biologically from their human counterparts; and in this model, pathogenic IgG binds platelets rapidly after injection, rendering IgG recycling pathways functionally irrelevant. In contrast, human chronic ITP is characterized by continuous autoantibody production and a substantial circulating IgG pool, which is more susceptible to modulation by FcRn blockade. Supporting this distinction, data from a phase 2 clinical trial showed that in approximately half of the patients who achieved a clinically meaningful platelet response, IgG levels were at their nadir at the time of response (48). These findings support FcRn as a clinically relevant therapeutic target in chronic ITP. While FcRn may not be universally required for all ITP interventions, it remains a promising target in therapies aimed at directly reducing the pathogenic IgG burden.

The development of FcRn inhibitors remains a rapidly evolving and expanding field. From the initial demonstration of efficacy in generalized Myasthenia Gravis to the current approval of multiple agents across a range of IgG-mediated disorders, we are witnessing remarkable progress. FcRn antagonists have introduced a paradigm-shifting therapeutic approach for antibody-driven autoimmune diseases, offering targeted efficacy through a unique mechanism of action. At the same time, the limitations and challenges associated with these therapies are increasingly recognized, highlighting the need for optimized drug design and refined clinical strategies. As more clinical trial data and mechanistic insights accumulate in the coming years, there is reasonable optimism that future generations of FcRn inhibitors will be safer, more effective, and more precisely tailored. Ultimately, the integration of FcRn-targeted therapies into personalized, multi-modal treatment regimens may significantly improve disease control and long-term outcomes in patients with refractory autoimmune diseases.
